# Assessment of Regional Ventilation During Recruitment Maneuver by Electrical Impedance Tomography in Dogs

**DOI:** 10.3389/fvets.2021.815048

**Published:** 2022-02-14

**Authors:** Aline Magalhães Ambrósio, Ana Flávia Sanchez, Marco Aurélio Amador Pereira, Felipe Silveira Rego Monteiro De Andrade, Renata Ramos Rodrigues, Renato de Lima Vitorasso, Henrique Takachi Moriya, Denise Tabacchi Fantoni

**Affiliations:** ^1^Department of Surgery, School of Veterinary Medicine and Animal Science, University of São Paulo, São Paulo, Brazil; ^2^Laboratory of Biomedical Engineering, Escola Politecnica, University of São Paulo, São Paulo, Brazil

**Keywords:** protective mechanical ventilation, atelectasis, overdistention, low tidal volume, oxygenation, stretch, respiratory mechanics

## Abstract

**Background:**

During protective mechanical ventilation, electrical impedance tomography (EIT) is used to monitor alveolar recruitment maneuvers as well as the distribution of regional ventilation. This technique can infer atelectasis and lung overdistention during mechanical ventilation in anesthetized patients or in the ICU. Changes in lung tissue stretching are evaluated by monitoring the electrical impedance of lung tissue with each respiratory cycle.

**Objective:**

This study aimed to evaluate the distribution of regional ventilation during recruitment maneuvers based on the variables obtained in pulmonary electrical impedance tomography during protective mechanical ventilation, focusing on better lung recruitment associated with less or no overdistention.

**Methods:**

Prospective clinical study using seven adult client–owned healthy dogs, weighing 25 ± 6 kg, undergoing elective ovariohysterectomy or orchiectomy. The animals were anesthetized and ventilated in volume-controlled mode (7 ml.kg^−1^) with stepwise PEEP increases from 0 to 20 cmH_2_O in steps of 5 cmH_2_O every 5 min and then a stepwise decrease. EIT, respiratory mechanics, oxygenation, and hemodynamic variables were recorded for each PEEP step.

**Results:**

The results show that the regional compliance of the dependent lung significantly increased in the PEEP 10 cmH_2_O decrease step when compared with baseline (*p* < 0.027), and for the nondependent lung, there was a decrease in compliance at PEEP 20 cmH_2_O (*p* = 0.039) compared with baseline. A higher level of PEEP was associated with a significant increase in silent space of the nondependent regions from the PEEP 10 cmH_2_O increase step (*p* = 0.048) until the PEEP 15 cmH_2_O (0.019) decrease step with the highest values at PEEP 20 cmH_2_0 (*p* = 0.016), returning to baseline values thereafter. Silent space of the dependent regions did not show any significant changes. Drive pressure decreased significantly in the PEEP 10 and 5 cmH_2_O decrease steps (*p* = 0.032) accompanied by increased respiratory static compliance in the same PEEP step (*p* = 0.035 and 0.018, respectively).

**Conclusions:**

The regional ventilation distribution assessed by EIT showed that the best PEEP value for recruitment maintenance, capable of decreasing areas of pulmonary atelectasis in dependent regions promoting less overinflation in nondependent areas, was from 10 to 5 cmH_2_O decreased steps.

## Introduction

Electrical impedance tomography (EIT) can infer atelectasis and lung overdistention during mechanical ventilation in both men and dogs during anesthesia and in the ICU (1). Experimental studies in dogs verify that EIT can identify pulmonary edema, changes in lung air and liquid volumes, lung hyperinflation, and effects of anesthesia and body positioning on EIT. Also a correlation of EIT and CT images was demonstrated in anesthetized dogs ventilated at different PEEP levels (1). Only one clinical study evaluated EIT during the recruitment maneuver (RM) in dogs undergoing sterilization surgery (2).

Therefore, EIT is a noninvasive and radiation-free imaging method that is used to monitor global and regional changes in lung volumes, aeration, ventilation, and heterogeneity (3). Global measurement of oxygenation and respiratory mechanics used to adjust mechanical ventilation may produce mistakes because the aeration may be different in the different regions of the lungs. Furthermore, there may be lung units being recruited and others overdistended during mechanical ventilation associated with RM. Heterogeneities in lung properties or pressure transmission caused by disease, aging, or gravity forces emphasize the side effects of mechanical ventilation (4). EIT is capable of quantifying regional ventilation in dependent and nondependent lungs to evaluate the silent space (SS) lung area and center of ventilation (CoV). Poorly ventilated lung units can mean atelectasis in gravity-dependent lung regions or overinflation in nondependent regions (3). Lung areas with impedance changes <10% of the maximum are defined with SS areas and can be used as a monitoring tool to guide lung protective ventilation during surgery. Similarly, CoV can estimate the air shift between the dorsal and ventral regions of the lungs during mechanical ventilation (5).

Protective ventilation has been studied in humans for several years in the ICU and anesthesia to promote less stretching of the pulmonary parenchyma in opposition to the high tidal volume used in the past. This strategy aims to reduce postoperative pulmonary complications using low tidal volume associated with RM and moderate PEEP levels (6, 7). The RM is recommended to reverse hypoxemia due to atelectasis during anesthesia and mechanical ventilation. The formation of atelectasis is expected due to the relaxation of the intercostal and abdominal muscles, diaphragm, and lungs after the administration of anesthetic or sedative drugs in addition to the atelectasis caused by the reabsorption of 100% FiO_2._ Atelectasis in dorsal-dependent lung areas and overdistention in ventral-independent lung areas are the main pathogenic mechanisms that produce a mismatch in the regional distribution of ventilation and perfusion that promotes gas exchange damage during general anesthesia (8). Meanwhile, RM by progressive increases in PEEP improved regional compliance of the dependent portions of the lung associated with a more homogeneous distribution of aeration. However, higher levels of PEEP were associated with lower compliance of nondependent regions of the lung, which could indicate overdistention. In healthy dogs in dorsal recumbency ventilated with low (7 mL.kg^−1^) and high (12 mL.kg^−1^) tidal volume (Vt), PEEP 5 cmH_2_O, and RM we observed that, at both Vt values, ventilation was poorly distributed to the dorsal dependent lung region before RM. However, after RM, the ventilation was centered more dorsally in the lung when the high Vt was employed (2). Nevertheless, this study did not demonstrate possible areas of atelectasis or hyperinflation. In horses, a study of the distribution of the aeration during alveolar RM and evaluation with EIT in general anesthesia with isoflurane in dorsal recumbency noted that the distribution of ventilation was displaced to the left dorsal direction during recruitment. Their results show that a proportion of Vt distributed to dependent lung areas increases during recruitment due to the opening of the atelectatic alveoli, but the occurrence of overinflation caused by RM was not evaluated (7).

Although several studies in dogs (7, 9–14) and horses (15–20) study alveolar RM with different tidal volumes and alveolar opening maneuvers, just one in horses studied the possible occurrence of overinflation (17).

Therefore, this study aimed to evaluate the distribution of regional ventilation during RM based on lung monitoring evaluation with EIT, during low Vt, focusing on better recruitment associated with less or no overdistention. We hypothesized that, during the descendent PEEP titration, there would be a decrease in areas of pulmonary atelectasis in the dependent regions without promoting overinflation in nondependent areas assessed by EIT.

## Materials and Methods

### Animals

The study was carried out at a veterinary teaching hospital linked to a faculty of veterinary medicine and animal science. The experimental protocol was approved by the institutional animal use ethics committee under protocol no. 9484170715 (11/2016).

In this study, seven dogs, five males and two females, weighing 25.0 ± 6.0 kg (mean ± SD), age 3.0 ± 1.0 years of different breeds, were investigated.

The inclusion criteria were nonobese dogs based on a body condition score of 4–6/9 according to the Laflamme scale (1997) and a thoracic circumference >60 cm due to the limited size of the EIT belt. Only clinically healthy animals with no history of respiratory or cardiovascular disease and no abnormalities on CT scans of the thorax, arterial blood gases, blood cell count, renal and liver plasma chemistry panel were included in the study.

These animals were subjected to ovariohysterectomy or orchiectomy, and client written informed consent was obtained before entering any dog into the present study protocol.

### Anesthesia and Monitoring

Food was withheld for 8 h and water for 4 h before anesthesia. The dogs were premedicated, intramuscularly (IM) with acepromazine (0.03 mg·kg^−1^; Syntec do Brasil, SP, Brazil) associated with meperidine (3 mg·kg^−1^; Dolosal; Cristália Produtos Químicos Farmacêuticos Ltda, SP, Brazil). After 15 min, a 20-gauge catheter (Angiocath; Becton Dickinson Indústrias Cirúrgicas, SP, Brazil) was placed in the left cephalic vein, and 5 mg·kg^−1^ propofol (Propovan; Cristália Produtos Químicos Farmacêuticos Ltda, SP, Brazil) was injected intravenously (IV) for induction of anesthesia. After orotracheal intubation, the animals were placed in dorsal recumbency on a heated mat and a blanket covering their bodies to avoid hypothermia. The endotracheal tube was connected to a microprocessor-controlled ICU ventilator, incorporating a calibrated variable orifice flow sensor pneumotachograph (G5; Hamilton Medical, Bonaduz, Switzerland). Anesthesia was maintained with continuous rate infusion (CRI) of propofol and remifentanil (0.2–0.4 mg·kg^−1^·min^−1^ and 0.2-0.4 μg·kg^1^·min^−1^, respectively) administered by an infusion pump (Agilia SP MC, Fresenius Kabi, Brazil). Neuromuscular blockade was initiated by IV administration of rocuronium (0.6 mg·kg^−1^; Esmeron; Organon do Brasil Indústria e Comércio Ltda, SP, Brazil) a bolus then continuously injected (1 mg·kg^−1^·h^−1^) to maintain mechanical ventilation with a peripheral nerve stimulator for assessment of the degree of the neuromuscular blockade (TOF- Guard Biometer; Organon Teknika, SP, Brazil) to ensure complete muscle paralysis associated with airway pressure curve and capnography. The animals were maintained in mechanical ventilation in the volume-controlled mode (7 ml·kg^−1^), ZEEP (zero end expiratory pressure) up to the first 30 min of anesthesia, inspiration-expiration ratio (I:E) of 1:2, FiO_2_: 100%, and the respiratory rate adjusted to maintain PE'CO_2_ between 35 and 50 mmHg (5-7 kPa). A nondispersive sidestream infrared gas analyzer (POET IQ2-8500; Criticare System Inc.) was used for airway gases measured and calibrated before each experiment, and PE'CO_2_ was measured.

### Experimental Protocol

The alveolar RM consisted of a series of PEEP increments of 5 cmH_2_O, beginning with 0 cmH_2_O up to 20 cmH_2_O followed by step decreases of 5 cmH_2_O and ending with a PEEP of 5 cmH_2_O. A 5-min interval between each PEEP level was allowed, and the whole maneuver lasted 35 min. At the beginning of the recruitment maneuver, the FiO_2_ was changed from 100% to 40% and maintained until the end of the anesthetic procedure.

### Data Collection

#### EIT Measurements

We used an EIT device (EIT Pioneer Set, Swisstom, Landquart, Switzerland), and all animals participated in the EIT protocol during mechanical ventilation. The EIT belt consisted of 32 equidistant electrodes, and it was placed around the animal's thorax. This belt was placed in the fifth and sixth intercostal spaces to prevent the formation of images of the heart or abdominal structures. The belt area was previously clipped and an ultrasound gel used to improve the contact of the electrodes with the skin. The EIT then evaluated the image acquisition cycle at a rate of 46 images *per second*, each cycle being acquired for 1 min and immediately archived on the computer and obtained off-line later. After the acquisition of all moments, the images were reconstructed with software (EIT Monitor STEM—Swisstom, Landquart, Switzerland) and directed by another specific software (IBex 1.5—Swisston, Landquart, Switzerland). The signal was filtered by ensemble averaging to remove cardiac-related impedance changes. Four breaths were analyzed at each time point for ventilation-induced impedance changes as previously described (2).

##### CoV

The CoV was calculated breath by breath from right to left (CoV R-L) along the *x*-axis and in the ventral to the dorsal direction (CoV V-D) along the *y*-axis, where *x* is the right-to-left distance of each pixel (zero is at the right lateral side) and *y* is the ventral–dorsal distance of each pixel (zero is at the ventral side). CoV was calculated as previously defined by the sum of the relative impedance changes related to ventilation per row of the tidal EIT images. The average of the resulting ventral-to-dorsal (0% 1/4 ventral; 100% 1/4 dorsal) CoV was plotted in a histogram over time to allow a view of potential changes in the ventilation distribution.

##### SS

The SS considered the area of the lung in which there was <10% difference in the impedance of the lung tissue (5). As a reference line, the line perpendicular to the gravity vector (measured by the sensor belt connector) running right through the center of ventilation is taken. This line is called a ventilation horizon and is used to sort the pixels of the lowest stretch category into dependent and nondependent SS. The SS value above the ventilation horizon was expressed as a percentage of the total number of pixels within the lung contour and called nondependent SS (NSS), and the value of the SS below the ventilation horizon was expressed as a percentage of the total number of pixels within the lung contour and is called dependent SS (DSS). “Dependent” in this context means located physically below a reference line within the thorax, whereas “nondependent” means above such a reference line within the thorax.

##### Tidal Volume Region of Interest (V_*TROI*_)

The V_TROI_ was generated from functional EIT images that were equally divided horizontally into four regions (from ROI 1 to ROI 4) covering the ventral-to-dorsal diameter (ROI 1 ventral; ROI 2 central-ventral; ROI 3 central-dorsal; ROI 4 dorsal). The fraction of tidal distribution of impedance signal in the ROI independent was defined as the sum of ROI 1 and ROI 2, and the ROI dependent was the sum of the ROI 3 and 4. Dependent (V_TROID_) and independent (V_TROIIn_) tidal volume were obtained by adding the tidal volume of the respective ROI (21).

##### Regional Compliance (Compl_*ROI*_)

Regional compliance for ROI dependent and independent lungs was calculated using the fraction of tidal distribution of impedance signal in the ROI dependent or independent, respectively (21).

##### Tidal Distribution Index (TDI)

The TDI was used to define the anteroposterior ventilation ratio and to assess the homogeneity of the tidal breath distribution. TDI closer to one implied more homogeneous ventilation by a shift of ventilation toward dependent regions (21).

#### Lung Mechanics, Gas Exchange, and Hemodynamics Data

A variable orifice flow sensor (Hamilton-G5, Hamilton Medical, Bonaduz, Switzerland) accurately measured the flow, volume, and pressure in the patient's airway and was calibrated before each patient's ventilation. The volume was calculated by the integration of the flow signal over time. The data were analyzed off-line, and static compliance (Cst) and drive pressure (DP) (22) were calculated. A multiparametric monitor system (Nihon Kohden Life Scope Triton—Nihon Kohden Corporation., Japan) was used for the continuous evaluation of electrocardiography, heart rate (HR), and mean arterial blood pressure (MABP). A 20-gauge catheter was placed in the dorsal pedal artery for pressure monitoring and collection of blood samples for measurement of pH, PaO_2_, and PaCO_2_, (ABL 330, Radiometer Medical ApS, Denmark).

#### Pulmonary Complications

Telephone contact with the owners of the dogs involved in the study was maintained by researchers for 10 days after anesthesia. Owners were asked about the occurrence of any signals that could be related to pulmonary complications.

### Statistical Analysis

Data were assessed for normality with the Shapiro–Wilk test before test selection and calculated and reported as median and IQR. A time influence was analyzed with one-way ANOVA followed by a *post-hoc* Tukey test to identify differences between time points. Statistical significance was attributed when *p* < 0.05. Analyses were performed using GraphPad Prism 6 (GraphPad Software). Sample size calculations (https://www.sealedenvelope.com) indicated six dogs were enough to have a 95% chance to observe a difference of 6% at a risk of 5% in the CoV V-D, considering 3% of standard deviation in the population. Data used for this calculation were extracted from a previous study (2).

## Results

### Animal Characteristics

A total of seven dogs were studied, weighing 25.0 ± 6.0 kg of body weight; 3 ± 1 year old; five males and two females; three dogs with no defined breed, one American Staffordshire terrier, one border collie, one basset hound, and one Labrador retriever. The chest circumference of these dogs was 68.0 ± 4.0 cm.

### Effects of RM on EIT Data

**Figure 1:**
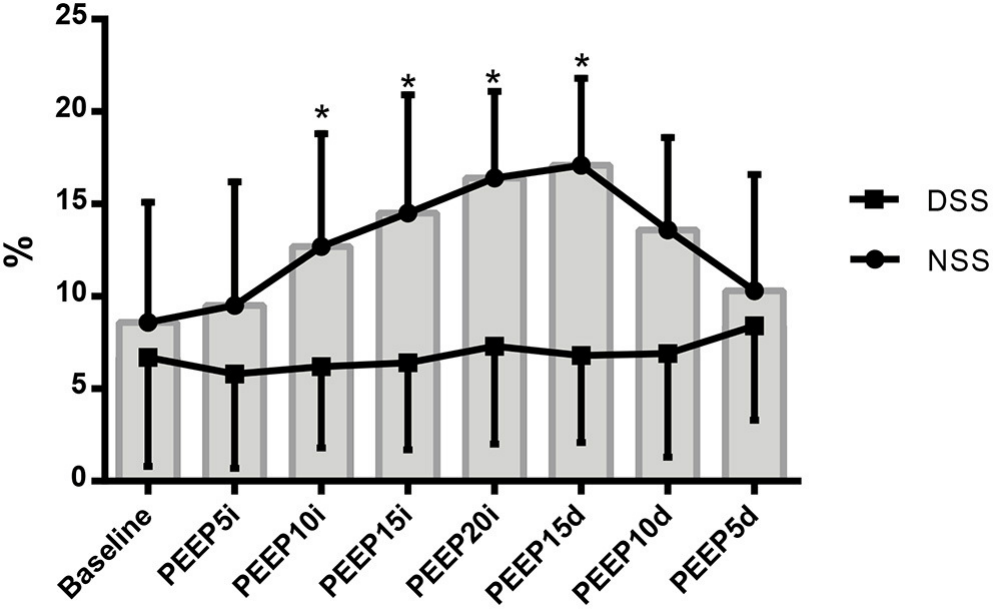
Variation of the NSS (closed circle) and DSS (closed square) regions of lung represented by mean ± standard deviation during recruitment maneuver for PEEP titration. NSS, nondependent silent space; DSS, dependent silent space. PEEP5i, PEEP 5 cmH_2_O increase; PEEP10i, PEEP 10 cmH_2_O increase; PEEP15i, PEEP 15 cmH_2_O increase; PEEP20i, PEEP 20 cmH_2_O increase; PEEP15d, PEEP 15 cmH_2_O decrease; PEEP10d, PEEP 10 cmH_2_O decrease; PEEP5d, PEEP 5 cmH_2_O decrease. *Statistically significantly different from baseline.

The effects of RM on EIT data are summarized in Table 1 and Figures 1, 3. There was no statistically significant change in CoV and TDI parameters during RM. Regional compliance of the dependent lung increased significantly by 75% in the PEEP 10 cmH_2_O decrease step compared with baseline (*p* < 0.027) although regional compliance of the nondependent lung decreased by 38% in the PEEP 20 cmH_2_O step (*p* = 0.039) compared with baseline.

**Table 1:** Electrical impedance tomography data.

**Variable**	**Baseline**	**PEEP5inc**	**PEEP10inc**	**PEEP15inc**	**PEEP20**	**PEEP15dec**	**PEEP10dec**	**PEEP5dec**
**CoVD-V (%)**	57.6 [50.5–59.1]	57.0 [52.6–60.7]	57.7 [54.9–59.7]	59.9 [58.2– 60.8]	60.7 [60.0–62.8]	61.0 [58.7–63.4]	59.2 [ 55.2–61.1]	55.5 [51.7–58.2]
**SSN (%)**	10.4 [0 −11.9]	10.4 [2.1–16.0]	12.9 [6.7–19.7]^*^	14.0 [8.8–20.7]^*^	16.0 [11.4–19.7]^*^	15.5 [13.5–20.2]^*^	13.5 [8.3–18.1]	11.4 [3.6–17,6]
**SSD (%)**	2.6 [2.6–14.0]	3.1 [2.6–10.9]	6.7 [2.1–9.8]	4.7 [2.6–11.9]	5.2 [3.6–14.5]	5.7 [3.1–11.9]	5.7 [1.5–13.5]	10.4 [3.6–13.5]
**Regional compliance ROI1 (ml/cmH**_**2**_ **O)**	1.7 [1.3–1.9]	1.2 [0.8–1.5] ^*^	0.7 [0.3–0.9]^*^	0.4 [0.2–0.6]^*^	0.4 [0.1–0.5]^*^	0.3 [0.1–0.5]^*^	0.3 [0.1–0.6]^*^	0.8 [0.2–1.5]^*^
**Regional compliance ROI2 (ml/cmH**_**2**_ **O)**	16.7 [3.4–18.1]	13.2 [5.9–15.79]	9.2 [7.5–11.8]	8.3 [6.5–9.3]	6.4 [5.2–7.8]	6.6 [5.8–8.6]	8.0 [6.8–9.3]	9.8 [9.2–10.9]
**Regional compliance ROI3 (ml/cmH**_**2**_ **O)**	22.7 [16.9–24.5]	23.3 [16.5–29.2]	23.7 [21.0–24.7]	21.9 [16.9–25.4]	20.4 [16.7–24.9]	21.5 [17.4–27.9]	21.2 [18.8–25.4]	21.2 [16.6–25.4]
**Regional compliance ROI4 (ml/cmH**_**2**_ **O)**	2.0 [1.1–5.8]	2.3 [1.2–5.3]	4.3 [3.2–6.5]	7.4 [3.9–8.2]	7.2 [4.0–9.4]	7.3 [3.7–9.8]	5.7 [3.2–8.1]	3.0 [1.7–5.9]
**Regional compliance non–dependent lung (ml/cmH**_**2**_ **O)**	4.8 [3.9–6.3]	5.2 [4.0–8.8]	4.1 [3.8–4.7]	3.4 [3.1–4.3]	2.6 [2.3–2.9]^*^	4.5 [3.2–4.5]	5.5 [4.3–6.0]	5.8 [5.3–7.4]
**Regional compliance dependent lung (ml/cmH**_**2**_ **O)**	6.2 [5.9–11.4]	8.1 [5.6–10.8]	8.7 [6.9–10.0]	9.0 [6.9–10.3]	6.5 [6.0–7.4]	9.8 [10.3–15.4]	11.1 [10.3–15.4]^*^	9.4 [9.0–14.2]
**Tidal distribution index**	0.53 [0.37–1.02]	0.70 [0.38–0.81]	0.51 [0.35–0.64]	0.43 [0.31–0.49}	0.35 [0.28–0.40}	0.36 [0.26–0.46]	0.46 [0.28–0.57]	0.58 [0.38–0.81]

*Data are expressed as median [IQR]*;

* *Statistically significantly different from baseline*.

*PEEP, positive end-expiratory pressure; PEEP5inc, PEEP 5 cmH_2_O increase; PEEP10inc, PEEP 10 cmH_2_O increase; PEEP15inc, PEEP 15 cmH_2_O increase; PEEP 20, PEEP 20cmH_2_O; PEEP15dec, PEEP 15 cmH_2_O decrease; PEEP10dec, PEEP 10 cmH_2_O decrease; PEEP5dec, PEEP 5 cmH_2_O decrease. CoVD-V, center of ventilation dorso-ventral; SSN, nondependent silent space; SSD, dependent silent space; ROI 1, region of interest ventral; ROI 2, region of interest centro-ventral; ROI 3, region of interest centro-dorsal; ROI 4, region of interest dorsal*.

ROI 1, which represents the most ventral and nondependent lung region, showed the highest potential for derecruitment because its compliance decreased significantly as the PEEP was increased and continued to fall until a maximum of 82% drop in PEEP 15 and 10 cmH_2_O (*p* = 0.001 and *p* = 0.003, respectively) decreased step. There were no significant changes in ROI 2, 3, and 4 compliances although ROI 4 regional compliance showed a high recruitment potential, more than 100%, in the PEEP 15 increase step, 20 cmH_2_O, and in the PEEP 15 cmH_2_O decreased step compared with baseline.

RM promoted an important increase in SSN values. A higher level of PEEP was associated with a significant increase in SSN from PEEP 10 cmH_2_O increased step (*p* = 0.048) until PEEP 15 cmH_2_O (0.019) decreased step, then it returned to close baseline values. The highest value was in PEEP 20 cmH_2_0, where it promoted a 53% increase (*p* = 0.016) even though the DSS did not show any significant changes.

### Effects of RM on Lung Mechanics, Gas Exchange, and Hemodynamics Data

**Figure 2:**
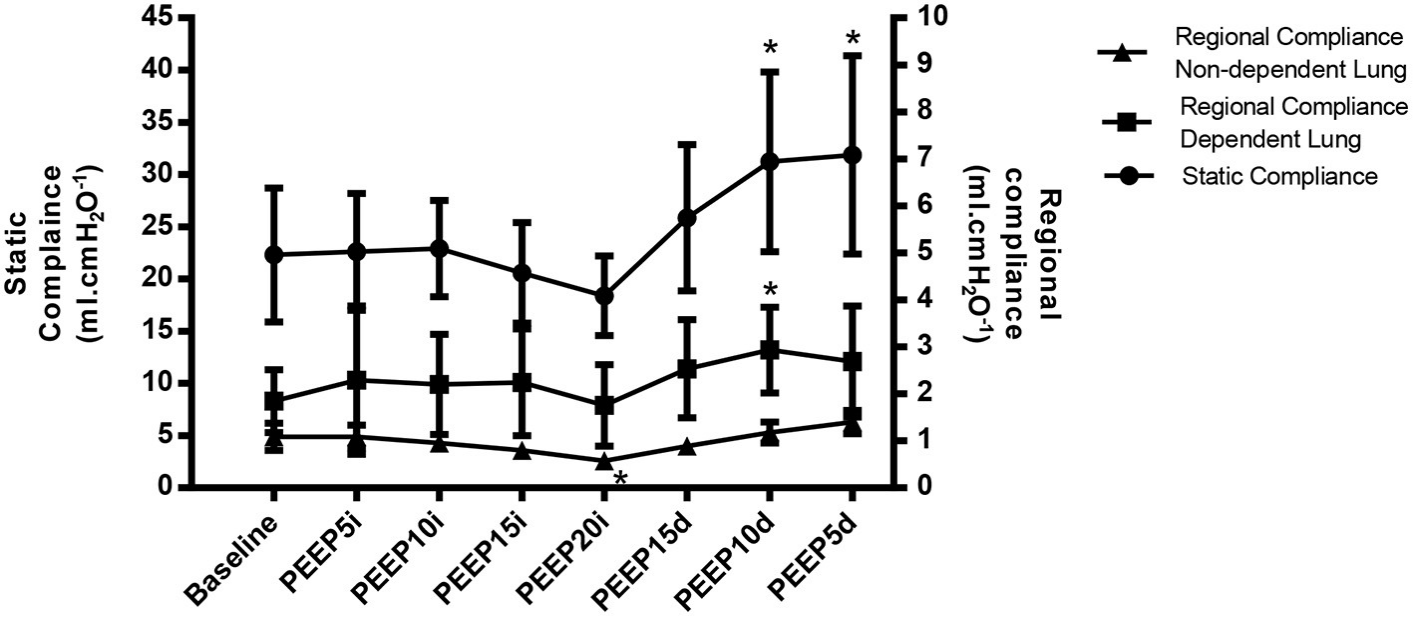
Variation of the global lung compliance (closed circle), regional compliance of nondependent lung (closed triangle) and regional compliance of dependent lung (closed square) represented by mean ± standard deviation during recruitment maneuver for PEEP titration. PEEP5i, PEEP 5 cmH_2_O increase; PEEP10i, PEEP 10 cmH_2_O increase; PEEP15i, PEEP 15 cmH_2_O increase; PEEP20i, PEEP 20 cmH_2_O increase; PEEP15d, PEEP 15 cmH_2_O decrease; PEEP10d, PEEP 10 cmH_2_O decrease; PEEP5d, PEEP 5 cmH_2_O decrease. *Statistically significantly different from baseline.

Lung mechanics, gas exchange, and hemodynamics data are summarized in Table 2 and Figure 2. Although PIP and Pplat increased and decreased as PEEP (*p* < 0.04), DP decreased significantly in the PEEP 10 and 5 cmH_2_O decreased steps (*p* = 0.032) accompanied by increased Cst in the same steps of PEEP (*p* = 0.035 and 0.018, respectively).

**Table 2:** Lung mechanics, gas exchange and hemodynamics data.

**Parameter**	**Baseline**	**PEEP5inc**	**PEEP10inc**	**PEEP15inc**	**PEEP20**	**PEEP15dec**	**PEEP10dec**	**PEEP5dec**
**V**_TE_ **(ml.Kg**^**−1**^**)**	6.96 [6.72–7.23]	6.80 [5.93–7.27]	6.62 [5.82–7.07]	6.89 [5.6–7.48]	7.07 [6.46–7.83]	6.78 [6.54–7.08]	6.78 [6.03–6.97]	6.86 [6.54–7.11]
**RR (mov.m** ^**−1**^ **)**	20 [15–25]	22 [15–25]	22 [15–25]	22 [20–32]	22 [20–34]	30 [22–34]	30 [22–34]	30 [22–34]
**PIP (cm.H**_**2**_ **O)**	9 [7.6–11.0]	13 [12–15] ^*^	19 [18–20] ^*^	24 [23–26] ^*^	32 [30–33] ^*^	23 [22–24] ^*^	17 [15–17] ^*^	12 [11, 12] ^*^
**Pplat (cm.H**_**2**_ **O)**	8 [6.7–9.0]	13 [8.0–14] ^*^	18 [14–19] ^*^	24 [19–25] ^*^	31 [25–31] ^*^	22 [19–23] ^*^	16 [14–16] ^*^	11 [8.5–11] ^*^
**DP (cm.H**_**2**_ **O)**	8 [6.7–9.0]	8 [7–9]	8 [7-9]	9 [7–10]	11 [10–12]	7 [6–8]	6 [5, 6] ^*^	6 [6] ^*^
**Cst (ml.cm.H**_**2**_ **O** ^**−1**^ **)**	20.9 [18.0–23.9]	22.6 [17.8–25.5]	22.9 [18.8–24.7]	20.9 [15.3–23.4]	18.9 [14.8–20.9]	22.0 [20.9–33.7]	30.1 [24.3–40.0] ^*^	31.5 [26.2–35.3] ^*^
**PaO**_**2**_ **/FiO**_**2**_	470.8 [439.−515]	450.0 [393–499]	426.8 [381–483]	442.8 [376–460]	475.0 [438–508]	465.5 [449–534]	452.8 [430–487.]	462.8 [410–496]
**PaCO**_**2**_ **(mmHg)**	47.8 [41.4–51.2]	48.1 [46.5–60.8]	53.2 [48.9–64.4]	57.3 [53.9–64.4] ^*^	55.4 [52.7–69.5]	57.6 [49.7–62.9]	57.2 [49.1–60.4]	53.8 [48.1–61.4]
**pH**	7.28 [7.26–7.34]	7.30 [7.28–7.31]	7.27 [7.21–7.29]	7.24 [7.19–7.26]	7.23 [7.22–7.27]	7.23 [7.22–7.26]	7.26 [7.20–7.20]	7.26 [7.18–7.28]
**MAP (mmHg)**	72 [61–82]	67 [63–74]	59 [57–81]	66 [60–77]	55 [42–83]	70 [52–77]	71 [60–89]	77 [65–91]
**HR (beats.min** ^**−1**^ **)**	91 [81–102]	100 [79–123]	120 [83–146]	145 [107–167] ^*^	150 [131–158] ^*^	150 [104–177] ^*^	141 [110–152] ^*^	117 [94–140]

*Data are expressed as median [IQR]*;

* *Statistically significantly different from baseline*.

*PEEP, positive end-expiratory pressure; PEEP5inc, PEEP 5 cmH_2_O increase; PEEP10inc, PEEP 10 cmH_2_O increase; PEEP15inc, PEEP 15 cmH_2_O increase; PEEP 20, PEEP 20 cmH_2_O; PEEP15dec, PEEP 15 cmH_2_O decrease; PEEP10dec, PEEP 10 cmH_2_O decrease; PEEP5dec, PEEP 5 cmH_2_O decrease. V_TE_, expiratory tidal volume; RR, respiratory rate; PIP, inspiratory peak pressure; Pplat, respiratory plateau pressure; DP, drive pressure; Cst, static compliance of respiratory system; PaO_2_/FiO_2_, ratio of arterial partial pressure of oxygen and oxygen inspiratory fraction; PaCO_2_, arterial partial pressure of carbon dioxide; pH, hydrogen concentration; MAP, mean arterial pressure; HR, heart rate*.

**Figure 3:**
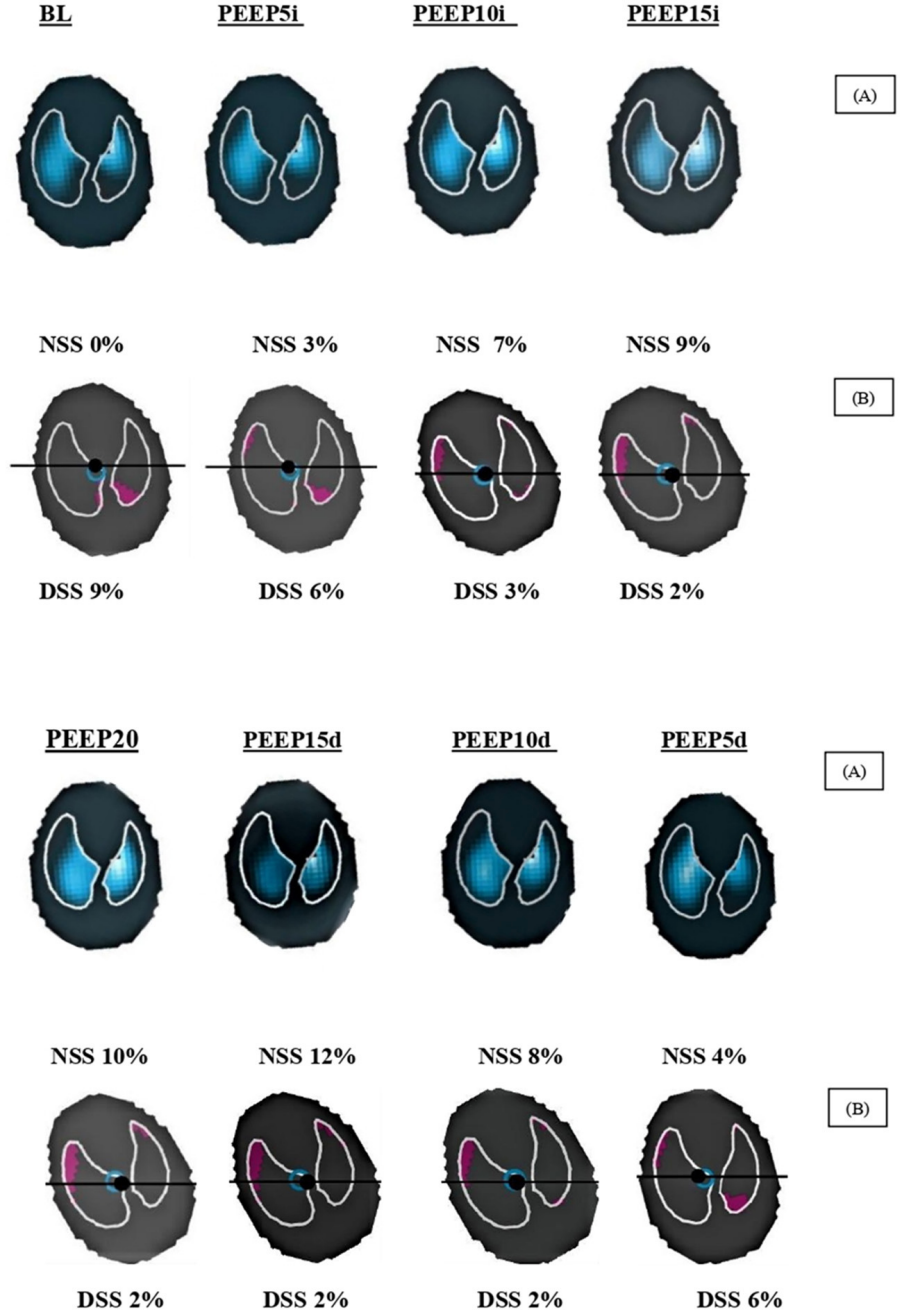
Regional impedance variation and DSS and NSS values of a representative dog during recruitment maneuver for PEEP titration. NSS, nondependent silent space; DSS, dependent silent space; BL, baseline; PEEP5i, PEEP 5 cmH_2_O increase; PEEP10i, PEEP 10 cmH_2_O increase; PEEP15i, PEEP 15 cmH_2_O increase; PEEP20i, PEEP 20 cmH_2_O increase; PEEP15d, PEEP 15 cmH_2_O decrease; PEEP10d, PEEP 10 cmH_2_O decrease; PEEP5d, PEEP 5 cmH_2_O decrease. **(A)** Tidal ventilation within the region of interest. The impedance changes (ΔZ) are larger in more light blue regions and the impedance changes are lower in more black regions. **(B)** SS is visualized as pink areas within the region of interest. The black line shows the ventilation horizon and the black dot represents the CoV. When increasing PEEP steps, the DSS decreased, and NSS increased, but the opposite was also true.

PaCO_2_ showed an increase significantly only at the PEEP 15 cmH_2_O increase step (*p* = 0.02) despite having maintained high values throughout the study. The pH maintained low values from the beginning of the study to the end but without significance between the evaluated moments.

HR showed values high, about 64% in the PEEP 15 cmH_2_O increase step (0.009), PEEP 20 cmH_2_O (0.008), PEEP 15 cmH_2_O (0.034), and PEEP 10 cmH_2_O (0.032) decrease step, but there was no statistically significant change in MABP and PaO_2_/FiO_2_.

No postoperative pulmonary complications were observed within 10 days of observation.

## Discussion

The main findings of the current study show that (1) RM by PEEP titration increased the regional compliance by 75% of the dependent lung at the PEEP 10 cmH_2_O decrease step. Furthermore, the EIT showed that, in the isolated analysis of the ROIs, although it did not present statistical significance, there was a trend toward increased compliance of more than 100% in the most dependent region of the lung, beginning with the PEEP 10 cmH_2_0 increase step until the end of the PEEP titration. (2) Global static compliance of the respiratory system, on the other hand, showed a significant increase of about 50% at PEEP 10 and 5 cmH_2_O in the decrease step. This improvement in compliance in the dependent lung was accompanied by a drop of 38% in compliance of the nondependent lung at PEEP 20 cmH_2_O and a decrease in regional compliance in the most ventral area of the lung in the highest PEEP. (3) NSS showed an increase from the PEEP 10 cmH_2_O increase step to the PEEP 15 decrease step. These findings corroborate with possible overinflation of the ventral region of the lung and seem to be related to high PEEP values. Ambrisko et al. (17) observed the same comportment in horses submitted to RM and conclude that increasing compliance at regional dependent areas following decreasing could mean recruitment and derecruitment of the dependent lung. On the other hand, increasing at nondependent regional compliance could mean overdistention, and decreasing could mean relief of then in the nondependent lung when the PEEP titration decreases too. According to Spadaro et al. (21), the balance between the recruitment of lung areas dependent on the gravity forces and the “acceptable” overdistention of nondependent regions can be found through the assessment of regional compliance, which is the most appropriate value of PEEP. Global respiratory mechanics can lead to frequent mistakes (21). In another recent study (5), the authors report that the measurement of SS seems to be a good tool to help clinicians balance and improve ventilation during anesthesia or at bedside because it allows better control of tidal volume, RM, and individualizing PEEP. TDI and CoV V-D were not able to detect significant changes in this study model although these variables represent a direction toward ventilation from nondependent to dependent regions of lungs. As demonstrated in the study by Spadaro et al. (21), the incremental PEEP level resulted in a significant reduction in TDI values closer to 1, mainly at PEEP 10 and 5 cmH_2_O decrease steps and change CoV toward 50% from PEEP 5 to 15 cmH_2_O increase step.

However, in this study, despite the low tidal volume, RM may have promoted overdistension in the higher PEEP levels. The decision to work with low values of tidal volume, 7 ml·kg^−1^ of body weight, to establish a protective ventilation protocol in dogs was based on the concept studied in man since 2013, where ventilation with low tidal volumes, PEEP, and RM, reduces the chance of causing mechanical ventilation-induced lung injury (VILI) in patients during general anesthesia as well as in patients with acute respiratory distress syndrome (ARDS) (23–25). According to Ball et al. (26), the main determinants of VILI are high pressure, high tidal volume, and cyclic alveolar opening and closing. Mechanical ventilation with high tidal volumes in man (10–15 ml·kg^−1^ of predicted body weight) shows evidence of causing alveolar overdistension, which promotes damage to the extracellular matrix and triggers an inflammatory process (26). In the present study, even a Vt of 7 ml·kg^−1^ was associated with areas of increased NSS pulmonary areas assessed by EIT during RM. These observations may suggest alveolar overdistension, especially during PEEP 20 cmH_2_O, even with maximum plateau pressure values of 30 cmH_2_O and volume below 10 ml·kg^−1^. In veterinary medicine, to our knowledge, no studies evaluate the occurrence of pulmonary overdistension with high volumes or the probability of long-term harm, that is, postoperative pulmonary complications, as they have already been widely demonstrated in man. Taking into account this fact, an important study, named Intraoperative Protective Ventilation (IMPROVE), prospective, controlled, and randomized with 400 patients undergoing major abdominal surgery, who received a ventilatory strategy consisting of low tidal volume, moderate levels of PEEP, and repeated MR to keep the lungs opened, compared with nonprotective ventilation, that is, with tidal volumes >10 ml·kg^−1^ predicted body weight and high PEEP, found that prophylactic protective ventilation reduced by 69% the occurrence of postoperative pulmonary complications in healthy lungs (23). According to Güldner et al. (27), after evaluating several studies on protective ventilation and the prevention of postoperative pulmonary complications, the use of low tidal volume was the most important factor to determine protection. Instead, a recent study that evaluated patients submitted to low (6 ml·kg^−1^ predicted body weight) or traditional tidal volume (10 ml·kg^−1^ predicted body weight) and fixed PEEP of 5 cmH_2_O in both groups during the intraoperative period did not show a significant reduction in pulmonary complications in 7 days postoperative (28).

The use of RM during general anesthesia is still being widely discussed in medicine, but current recommendations are that, for uninjured lungs, the maneuver should be performed with PEEP titration at low tidal volume and when peripheral O_2_ saturation is below 92% (27). Titration of PEEP on ascent aims to recruit collapsed alveoli and on the descent to establish the best PEEP point to keep the alveoli that were recruited opened and then using the “open lung concept,” already discussed by Lachmann (29). In the current study, we used RM to reverse atelectasis formed by anesthetic induction although oxygen saturation in arterial blood was on average 95%. According to Staffieri et al. (30), dogs ventilated with 100% FiO_2_, also in the supine position and weighing an average of 25 kg of body weight, despite maintaining a peripheral oxygen saturation of 99%, had more areas of atelectasis in the dependent lung regions observed on CT, thus demonstrating the importance of atelectasis formation by resorption in this species. In humans, atelectasis is formed from the induction of anesthesia and can last for hours in the postoperative period, resulting in hypoxemia in this period (31, 32). In dogs, Alwood et al. and Brainard et al. (22, 33) also report hypoxemia as an important postoperative complication after abdominal surgery under general inhaled anesthesia with 100% O_2_ without preexisting lung disease. Canfrán et al. (34) carried out a study of hypoxemia reversal in dogs using stepped RM and observed an improvement in oxygenation and compliance up to 60 min after the end of the maneuver and maintenance with a PEEP of 5 cmH_2_O. De Monte et al. (8) demonstrate a decrease in atelectasis in dogs through chest CT after 40 min of mechanical ventilation with a tidal volume of 12 ml·kg^−1^, FiO_2_ of 1.0 in ZEEP, after performing RM followed by reduction of FiO_2_ to 0.4 or maintaining only a PEEP of 5 cmH_2_O even with a FiO_2_ of 1.0. Garcia-Sanz et al. (9) did not observe an increase in arterial oxygenation in healthy dogs early after RM. Likewise, in the current study, we could not observe improvement in both arterial oxygen saturation and PaO_2_/FiO_2_ as these were already within normal values at the beginning of recruitment as they were lungs without previous injury. In healthy lungs, the percentage of potentially recruitable alveoli is very close to zero when compared with previously injured lungs because they have the normal functioning of the surfactant, which keeps the alveolar units in a noncollapsed state. According to Bugedo et al. (33), studies show that recruitment is more efficient in injured lungs with poor gas exchange and deteriorated pulmonary mechanics.

The ideal PEEP capable of keeping the alveoli open after RM is also not well defined in medicine, and current recommendations are to use low PEEP or, better yet, individualize it for each patient and specific surgical procedure (27). In veterinary medicine, the same fact also occurs, and studies in dogs usually apply a PEEP of 4 to 5 cmH_2_O for a bodyweight of 25 to 30 kg (2, 8). In the present study, the best compliance occurred between the descending PEEP of 10 and 5 cmH_2_O, and the best PEEP was between 10 and 5 cmH_2_O decrease step too.

According to data from the current study, the increases in peak and plateau pressure were significant at all times compared with baseline, but this was already expected due to the increase in inspiratory pressure previously determined in the RM by PEEP titration. The maximum value of PIP and Pplateau observed was 32 cmH_2_O at the maximum PEEP and reaching a maximum DP of 11 cmH_2_O. In humans, studies report greater importance in the DP values, that is, in the difference between the plateau pressure and the PEEP used, as much in ARDS-treated lungs as in healthy lungs (22, 34, 35). This variable is considered one of the most important factors in the pulmonary protective strategy today, together with the low tidal volume, low plateau pressure, and high-to-moderate PEEP values. Bugedo et al. (33) suggest the use of V_T_ between 6 and 8 ml.kg^−1^, moderate levels of PEEP, adjusting it according to the DP, which should be below 15 cmH_2_O in the injured lungs. The decrease in DP after the increase in PEEP reflects the recruitment and decrease in the cyclical stretching of the lungs during mechanical ventilation. On the other hand, an increase in DP suggests a not recruitable lung, in which overdistension prevails instead of alveolar recruitment. Bugedo et al. (33) also suggest that, if, after PEEP optimization, the DP remains above 15 cmH_2_O, the tidal volume should be decreased. Patients without ARDS likely have a DP below 10 cmH_2_O, reflecting a normal or near-normal static compliance of the respiratory system (Csr). As Crs is the quotient between V_T_ and DP (Pplateau—PEEP), the lower the DP, the higher the Csr (22). In contrast, in patients with moderate ARDS or restrictive lung diseases (pulmonary edema, pleural effusion, interstitial disease, fibrosis, etc.), the DP will be >10 cmH_2_O, which may reflect low Csr or inadequate V_T_/PEEP (33). The findings of the current study are in line with those of Bugedo et al. (33) as the DP started at 8 cmH_2_O before the RM, reaching a value of 11 cmH_2_O only in the PEEP of 20 cmH_2_O at the same time worsening of static compliance was verified. However, after RM, the DP decreased significantly to 6 cmH_2_O at PEEP 10 and 5 cmH_2_O descending, reflecting the improvement at 45% in the static compliance of the respiratory system (Cst) compared with baseline. These results may suggest that the best PEEP to maintain pulmonary recruitment in the current study was between 10 and 5 cmH_2_O descending because, at these times, the best respiratory system compliance and the lowest DP were obtained.

During RM, a significant increase in PaCO_2_ was observed at the PEEP 15 cmH_2_O increase step, following a consequently not significant drop in arterial pH. According to De Monte et al. (8), the increase in PaCO_2_ during RM can be explained by the reduction in alveolar CO_2_ and increased functional residual capacity, which consequently decreases its elimination as PEEP tends to reduce the amount of volume that circulates within the alveoli, increasing the anatomical dead space. Furthermore, venous return is reduced due to increased intrathoracic pressure, thus maintaining CO_2_ produced by tissues in the periphery and thus increasing its concentration in the blood (36). The drop in arterial pH suggests respiratory acidosis due to the increase in PaCO_2_, but this occurrence is expected during RM, being usually transitory with the pH returning to normal values after a few minutes. Oura et al. (37), studying different tidal volumes in beagles, reported that a tidal volume of 6 ml.kg^−1^ was efficient in ventilating these animals, even promoting mild hypercapnia and respiratory acidosis. Corroborating this fact, it is important to remember that, during protective ventilation, in which low tidal volumes are used, associated with RM and PEEP, hypercapnia is allowed due to its sympathomimetic effect in increasing HR and BP, which can minimize the cardiovascular depressive effect of positive pressure ventilation Laffey et al. (38) report that, in critically ill patients, both children and adults, a pH of up to 7.15 is well-tolerated.

Regarding the hemodynamic variables in the current study, only a significant increase in HR was observed from the PEEP 15 cmH_2_O increase step to the PEEP 15 cmH_2_O decrease step. The return of HR values closes to baseline occurred in the descending phase of RM, and no alteration in blood pressure was noticed. Normally, during RM, an increase in HR and a decrease in SBP, MAP, DBP, and CO is expected, especially at the higher PEEP values, but returning to normal as soon as the high intrathoracic pressures are removed. Corroborating these findings, Costa Lame et al. (6), studying the effects of alveolar recruitment and protective ventilation in patients undergoing cardiac surgery, found a transient decrease in blood pressure values during RM, but they returned to baseline 5 min after the end of the maneuver. Mercado et al. (39), studying the hemodynamic effects of alveolar RM through echocardiography, observed that, at higher PEEP values, systolic blood pressure and cardiac output decrease but return to normal values as soon as PEEP is decreased for values close to basal. These hemodynamic changes that occur during RM are explained by the decrease in right ventricular preload and increase in right ventricular afterload due to high mediastinal pressure and high airway pressure with increased pulmonary vascular resistance. Consequently, there is a reduction in the transmural pressure of the right atrium and superior vena cava and a decrease in cardiac output (40). However, this effect is more pronounced in hypovolemic patients than in normovolemic and hypervolemic patients, as demonstrated by Nielsen et al. (41) in an experimental study in pigs. In the current study, no significant drop in BP was observed, probably due to the compensatory increase in HR and probably because there was no hypovolemia. Thus, there was no need for fluid challenge and not even vasoactive drugs were administered during RM. Although there is acceptance of the concept of the need for its administration, it is important to remember that the hemodynamic effects of RM are closely related to the recruitment method and the level of alveolar pressure applied as well as the basic cardiovascular properties, in addition to the mechanics of the lungs and rib cage (41). According to Lim et al. (42), RM depresses CO only transiently, and the PEEP value influences the final effect on it.

The study animals were clinically reevaluated 10 days after the procedure and during this period contact by phone was maintained with their owners. However, none of the animals showed pulmonary complications within this period. According to Güldner et al., Hendestierna and Lannart (27, 43) the most important aspect of protective mechanical ventilation is the low tidal volume. Although Karalapillai et al. (28) evaluated low tidal volume (6 ml·kg^−1^) compared with conventional tidal volume (10 ml·kg^−1^), both with PEEP 5 cmH_2_O without RM undergoing major surgery do not observe the difference in postoperative pulmonary complications. Still, Costa Leme et al. (6), in cardiac surgery using low tidal volume (6 ml·kg^−1^) and comparing an intensive vs. moderate RM, observed less severe postoperative pulmonary complications (hypoxemia) with intensive RM.

One limitation of this study was that RM evaluation was carried out in healthy lungs, a fact that reduced the observation of its effects on improving lung oxygenation. Another limitation was that, although no postoperative pulmonary complications were observed within 10 days of the study, further studies should be carried out to evaluate for a longer period.

In conclusion, the distribution of regional ventilation assessed by EIT showed that the best PEEP value for RM maintenance, capable of decreasing pulmonary atelectasis areas in severely dependent regions promoting less overinflation in nondependent areas, was from 10 to 5 cmH_2_O decreased step for dogs in this study model. It was confirmed considering the best compliance and DP, the smallest range of overdistension, and the absence of hemodynamic changes in the same PEEP steps. Furthermore, no postoperative pulmonary complications were observed within 10 days of observation.

## Data Availability Statement

The raw data supporting the conclusions of this article will be made available by the authors, without undue reservation.

## Ethics Statement

The animal study was reviewed and approved by Ethics Committee on Animal Use of the School of Veterinary Medicine and Animal Science (University of São Paulo). Written informed consent was obtained from the owners for the participation of their animals in this study.

## Author Contributions

AA: designed the study, performed experimental procedures, interpreted the data, performed the statistics, and wrote the manuscript. AS, MP, FA, and RR: participated in study design and experimental procedure. RV and HM: interpreted the EIT data and critically reviewed the manuscript. DF: designed the study, performed experimental procedures, and critically reviewed the manuscript. All authors contributed to the critical revision of the paper and approved the final manuscript.

## Funding

This study was supported by a grant from the Fundação de Amparo a Pesquisa do Estado de São Paulo (FAPESP: 2015/14721-7).

## Conflict of Interest

The authors declare that the research was conducted in the absence of any commercial or financial relationships that could be construed as a potential conflict of interest.

## Publisher's Note

All claims expressed in this article are solely those of the authors and do not necessarily represent those of their affiliated organizations, or those of the publisher, the editors and the reviewers. Any product that may be evaluated in this article, or claim that may be made by its manufacturer, is not guaranteed or endorsed by the publisher.
